# The role of personality in the thoughts, feelings, and behaviors of students in Germany during the first weeks of the COVID-19 pandemic

**DOI:** 10.1371/journal.pone.0242904

**Published:** 2020-11-30

**Authors:** Eva Asselmann, Lex Borghans, Raymond Montizaan, Philipp Seegers

**Affiliations:** 1 Department of Psychology, Humboldt-Universität zu Berlin, Berlin, Germany; 2 Maastricht University, School of Business and Economics, Maastricht, The Netherlands; 3 CASE Candidate Select, Köln, Germany; Middlesex University, UNITED KINGDOM

## Abstract

We examined how the thoughts, feelings, and behaviors of 6,957 students from Germany, assessed between March 16 and April 21, 2020, when COVID-19 became a serious health concern in Germany, varied by personality. The Big Five personality traits—openness to experience, conscientiousness, extraversion, agreeableness, and emotional stability—were assessed with the International Personality Item Pool. Students were asked whether they kept up with the COVID-19 news, followed specific governmental rules and recommendations (washing hands more, using public transport less, avoiding larger crowds, and restricting meetings with family/friends), hoarded supplies, felt less secure in public places, or expected financial losses due to the crisis. Logistic regressions adjusted for sociodemographic factors and cognitive abilities revealed that more conscientious (odds ratio (OR) = 1.133) and more agreeable (OR = 1.285) students kept up with the news more. More agreeable students were also more likely to wash their hands more often/intensively (OR = 1.262), use public transport less (OR = 1.182), avoid crowds (OR = 1.320), and restrict meetings with family/friends (OR = 1.410). Other Big Five traits were not associated with these behaviors, except that less emotionally stable individuals tended to use public transport less often (OR = 1.162). Additionally, less emotionally stable students, in particular, more often bought more supplies than usual (OR = 1.322), felt insecure in public spaces (OR = 1.597), and expected financial losses (OR = 1.270). Moreover, less open (OR = 0.876) and more conscientious (OR = 1.235) students more often felt insecure in public spaces, and more extraverted individuals more often expected financial losses (OR = 1.180). Taken together, our findings suggest that more agreeable individuals, in particular, tend to comply with governmental rules and recommendations to fight COVID-19, whereas less emotionally stable individuals, in particular, tend to hoard supplies, feel insecure, and fear financial losses due to the crisis.

## Introduction

In March 2020, public health measures were taken in many countries to prevent the further spread of COVID-19. At the time, people suddenly had to adjust to a completely new situation. Now, several months later, we know that especially younger individuals sometimes engaged in potentially problematic behaviors at the beginning of the pandemic (e.g., mass events or parties) [1]. How did young adults think, feel, and behave during the first weeks of the COVID-19 pandemic? Psychological research suggests that individuals can differ considerably in their ways of handling this challenging new situation [2] and that these individual differences could be partially explained by the Big Five personality traits openness to experience, conscientiousness, extraversion, agreeableness, and emotional stability [3–10]. Investigating the role of the Big Five personality traits in students’ thoughts, feelings, and behaviors during the first weeks of the COVID-19 pandemic is crucial not only for our understanding of human behavior, but also for applied research on targeted interventions to fight COVID-19 and associated unfavorable consequences [11–13].

### Openness to experience

Open individuals tend to seek out new and unconventional ideas and experiences and to be flexible, curious, and creative [14–16]. During the COVID-19 pandemic, more open individuals could therefore be more likely to be interested in what is going on, to have fewer difficulties adjusting to the new situation, and to find more unconventional ways of coping with it.

### Conscientiousness

Conscientious individuals tend to be responsible, dutiful, and self-disciplined [14,17]. Therefore, highly conscientious individuals, in particular, could strictly follow governmental rules and recommendations to fight the virus and change their behavior accordingly.

### Extraversion

Extraverted individuals tend to be outgoing and sociable [14]. Therefore, more extraverted individuals could have greater difficulties eliminating social contacts and activities, avoiding crowds, and staying away from public places and activities. Consequently, more extraverted individuals could engage less strictly in social distancing during the COVID-19 pandemic.

### Agreeableness

Agreeable individuals tend to be kind, cooperative, and respectful [14]. During the COVID-19 pandemic, more agreeable individuals could thus comply more strictly with socially desirable safety rules and strive to support and protect others to maintain positive relationships with them. To avoid deviations from social norms and expectations, they could also pay stronger attention to other people’s behavior during these challenging times and adjust accordingly.

### Emotional stability

Less emotionally stable individuals tend to be more susceptible to stress and negative emotions such as depression and anxiety [14,18]. Therefore, less emotionally stable individuals could feel more distressed, depressed, anxious, and burdened during the COVID-19 pandemic. For example, they could be more disturbed by the new situation, be more cautious, and tend more toward safety and avoidance behavior. Less emotionally stable individuals might not only follow the governmental rules more strictly, but also take additional private measures (e.g., hoarding supplies) to stay safe, avoid potential harm, and fight their fear.

### Previous findings concerning the role of personality during the COVID-19 pandemic

Only a few previous studies have examined the role of the Big Five personality traits for specific thoughts, feelings, or behaviors during the COVID-19 pandemic [3–9]. A recent study among 2,066 adults (aged between 18 and 98 years) from the United States examined associations of the Big Five personality traits with financial concerns, containment measures (e.g., washing hands), and hoarding supplies in early March 2020 [4]. It found that more open individuals engaged more in containment measures and hoarded food more often. More conscientious individuals were less concerned about their financial situation and engaged more in containment measures. More extraverted individuals reported higher financial concerns, were more strongly engaged in containment measures, and hoarded supplies more often. More agreeable individuals reported lower financial concerns and engaged more in containment measures. Finally, less emotionally stable individuals reported higher financial concerns and engaged less in containment measures.

Another study investigated the role of conscientiousness and extraversion in social distancing and washing hands among 715 adults (aged between 18 and 78 years) from Brazil [3]. The findings suggest that more conscientious individuals engaged more in social distancing and washed their hands more, whereas more extraverted individuals were less compliant in terms of social distancing.

These previous studies covered the entire adult life span. As the COVID-19 pandemic has evolved, it has become clear that younger people, in particular, sometimes tend to underestimate the potential threat of COVID-19 and insufficiently comply with governmental rules and recommendations [1]. Moreover, recent findings suggest that younger individuals, in particular, tend to suffer from loneliness and mental health issues due to lockdown measures and social distancing [19,20]. Finally, younger adults (e.g., students) are oftentimes about to apply for jobs, start working life, and establish a career. Thus, they might perceive the consequences of COVID-19 on the economy and job market as particularly harmful [21]. Taken together, additional research is needed to focus particularly on younger people’s thoughts, feelings, and behaviors in spring 2020, when COVID-19 became a serious health concern around the globe.

### Aims

The aim of this study is to investigate associations between the Big Five personality traits and students’ thoughts, feelings, and behaviors during the first three weeks of the COVID-19 pandemic in Germany. We studied about 7,000 students in Germany from March 16 until April 21, 2020, approximately the time when the government in Germany introduced public health measures to prevent further spread of the disease.

Health regulation in Germany takes place at the state level. Therefore, different German states created slightly different regulations. From March 16 onward, most schools and universities in Germany closed to prevent further spread of COVID-19. Additional measures were taken a week later: people were only allowed to meet with a maximum of one other person or with people from their own household. People were recommended to avoid larger groups, keep a distance of 1.5 meters from others, and go to work/use public transport only when absolutely necessary.

We collected data on the Big Five personality traits and cognitive abilities. Moreover, participants indicated whether they (a) kept abreast of the news, (b) complied with governmental rules and recommendations, and (c) hoarded supplies, felt less secure, or feared financial losses due to the crisis.

### Hypotheses

Our hypotheses (with respect to each individual behavior and trait; see Table 1) were preregistered at https://osf.io/w5nqz. Our hypotheses were as follows. More open and more agreeable students are more likely to keep abreast of the news. More conscientious, more agreeable, and less emotionally stable students are more likely to comply with official rules and recommendations. Less emotionally stable students are more likely to hoard supplies, feel less secure, and fear financial losses due to the crisis.

**Table 1:** Hypothesized associations between the Big Five personality traits and students’ thoughts, feelings, and behaviors during the first three weeks of the COVID-19 pandemic in Germany.

		O	C	E	A	Low ES
1	I consciously keep an eye on the news situation	+			+	
2	I wash my hands more often/intensively		+		+	+
3	I use public transport less often/not at all		+		+	+
4	I avoid (larger) crowds		+	−	+	+
5	I restrict meetings with family/friends as a precaution		+	−	+	+
6	I have bought more supplies than usual				−	+
7	I feel more insecure in public space than usual					+
8	I expect financial losses (e.g. due to loss of my student job)	−		+		+

*Note*. O = Openness; C = Conscientiousness; E = Extraversion; A = Agreeableness; ES = low emotional stability.

## Data and methodology

### Study

We used data from the Fachkraft 2030 survey [22], which aims to measure the life situation and expectations of students in Germany from a general and economic perspective. The survey targets students at research universities (*Universität*) and universities of applied science (*Fachhochschule*) in Germany. The study is being carried out by one of the authors (Philipp Seegers) in collaboration with Studitemps, which invites all students of its very large job network, JobMensa, to participate. Responses from about 325,000 students in 16 waves since 2012 have been collected so far.

The survey is a repeated cross-sectional study conducted twice a year, in March and September. The survey focuses on life circumstances and expectations. In the end, students can participate in a psychological assessment that includes measurements of the Big Five personality traits and cognitive abilities. In this study, we used data from the 16th wave of the online Fachkraft 2030 survey, conducted between March 16 and April 21, 2020 (with most of the responses recorded between March 16 and April 9, 2020).

In the past, no systematic differences between participants of the Fachkraft 2030 survey and the German student population (as reported by the German Federal Statistical Office, Destatis) could be found, except that women were overrepresented by roughly 10 percentage points [22]. This overrepresentation of women is similar to that in another large study among students (*Sozialerhebung*) funded by the German government [23]. Compared to that study, the Fachkraft 2030 survey comprises a similar proportion of employed students, an important observation, given that the students are invited to participate through JobMensa.

All previous publications related to the survey, including yearly reports of the data, can be found at www.fachkraft2030.de, which links to the Studitemps website. All publications include a detailed description of the data in comparison to the demographics of the German student population.

### Assessment of thoughts, feelings, and behaviors due to the crisis

The 16th wave of the Fachkraft 2030 survey was conducted when the COVID-19 pandemic had became a serious national health concern in Germany. Therefore, participants of this wave were asked how the COVID-19 pandemic crisis affected their thoughts, feelings, and behaviors in everyday life. Specifically, the participants were asked to answer eight statements with either yes or no. One item captured whether participants kept an eye on the news situation (“I consciously keep an eye on the news situation”). Four items assessed whether participants complied with official rules and recommendations (“I wash my hands more often/intensively”; “I use public transport less often/not at all”; “I avoid (larger) crowds”; “I restrict meetings with family/friends as a precaution”). Finally, three items measured whether participants hoarded supplies (“I have bought more supplies than usual”), felt less secure (“I feel more insecure in public space than usual”), or feared financial losses (“I expect financial losses; e.g. due to loss of my student job”) due to the crisis.

### Assessment of the Big Five personality traits

The Big Five personality traits—openness to experience, conscientiousness, extraversion, agreeableness, and emotional stability—were assessed with the International Personality Item Pool (IPIP; S1 Table) [24]. The IPIP consists of 50 items with different statements describing the participants. The participants had to indicate their agreement with these descriptions, using a scale of one (“very inaccurate”) to five (“very accurate”). Example items are “Have a vivid imagination” for openness to experience, “Pay attention to details” for conscientiousness, “Talk to many different people at parties” for extraversion, “Sympathize with others’ feelings” for agreeableness, and “Am easily disturbed” for low emotional stability. In the sample considered in this paper, internal consistencies were α = 0.86 for openness to experience, α = 0.83 for conscientiousness, α = 0.76 for extraversion, α = 0.85 for agreeableness, and α = 0.75 for emotional stability.

### Assessment of cognitive abilities

Cognitive abilities were assessed with 27 Raven’s Progressive Matrices. Each respondent received six questions from this set, including three of 15 relatively easy items and three out of 12 relatively difficult items, chosen randomly (the difficulty of each item was determined based on previous waves). We applied Item Response Theory (IRT) with a two-parameter logistic parametrization (using Stata 15.1) to create a measure of cognitive ability.

### Statistical analysis

Using logistic regressions, we analyzed the associations (Odds Ratios, OR) of the Big Five personality traits (standardized predictors with Mean zero and SD = 1) with specific thoughts, feelings, and behaviors during the first weeks of the COVID-19 pandemic (with binary outcomes 1 = yes and 0 = no). Separate models were built for each behavioral outcome. The Big Five personality traits were included simultaneously as multiple predictors. The analyses were adjusted for cognitive abilities (standardized score), linear age (centered score with a mean of zero), and quadratic age (squared term of the linear age variable), as well as gender (female, male, and diverse). In line with non-binary gender concepts, the category ‘diverse’ was assessed to account for diverse gender identities that are not exclusively masculine or feminine. Cognitive abilities were included as a control variable because higher cognitive abilities have been associated with (a) higher health literacy and adherence to (public) health measures [25], as well as with personality, especially greater openness [26]. Not only linear but also quadratic age was included to account for nonlinear (i.e., U-shaped) age effects.

Our study was conducted at the early stage of the COVID-19 pandemic in Germany. Because participants could have completed the survey slightly before, during, or after specific governmental rules and recommendations were introduced, we repeated the analyses and additionally controlled for timing effects (i.e., by including a dummy for each day of answering the survey).

The alpha level was set at 0.05. To control for multiple testing, we applied the by-method of Benjamini and Hochberg [27].

## Results

### Sample characteristics

The Fachkraft survey consists of a short and a long version. After a short survey with basic questions, the participants are invited to take part in a second part that includes psychological measures. Overall, 15,330 students took part in the 16th wave of the online Fachkraft 2030 survey, of whom 7,219 students participated in the personality assessment. Of these individuals, 64 did not provide full information on their personality, another 24 did not complete Raven’s Progressive Matrices, and 174 did not provide full information on their demographics, leaving a final sample of 6,957 students for the analyses. Of these, 4,267 (61%) were female, 2,641 (38%) were male, and 49 (0.7%) identified as being diverse. The mean age of the sample was 23.93 years (SD = 3.63 years; range: 15 to 34 years).

### Frequencies of specific thoughts, feelings, and behaviors during the first weeks of the COVID-19 pandemic

Of the total sample, 4,313 individuals (62%) indicated they consciously were keeping up with the news. Furthermore, the majority indicated complying with governmental rules and recommendations to prevent further spread of the virus: 4,661 (67%) washed their hands more often/intensively than usual, 3,965 (57%) used public transport less or not at all, 4,940 (71%) avoided crowds, and 4,731 (68%) restricted meetings with family or friends as a precaution. Finally, 1,183 (17%) bought more supplies than usual, 1,948 (28%) felt more insecure in public spaces, and 2,783 (40%) expected financial losses due to the crisis.

### The role of personality

Table 2 shows the associations between the Big Five personality traits and specific thoughts, feelings, and behaviors during the first weeks of the COVID-19 pandemic, adjusted for cognitive abilities, age, and gender, which are summarized in Table 3 (equivalent to Table 1). The correlations between all of these variables are presented in S2 Table.

**Table 2:** Associations between the Big Five personality traits and students’ thoughts, feelings, and behaviors during the first three weeks of the COVID-19 pandemic in Germany.

(1) I consciously keep an eye on the news situation				
Predictor	*OR*	*SE*	*p*	*p*_*cor*_
Openness	0.959	0.048	0.402	0.536
Conscientiousness	1.133	0.053	0.008	0.018
Extraversion	1.018	0.043	0.665	0.739
Agreeableness	1.285	0.058	<0.001	<0.001
Low emotional stability	1.043	0.044	0.322	0.460
Cognitive ability	1.117	0.043	0.004	0.009
Age	1.723	0.136	<0.001	<0.001
Age^2^	0.988	0.002	<0.001	<0.001
Gender: female (vs. male)	0.957	0.055	0.441	0.569
Gender: diverse (vs. male)	0.860	0.262	0.620	0.709
Gender: diverse (vs. female)	0.899	0.273	0.725	0.773
(2) I wash my hands more often/intensively				
Predictor	*OR*	*SE*	*p*	*p*_*cor*_
Openness	0.887	0.046	0.021	0.046
Conscientiousness	1.010	0.049	0.830	0.863
Extraversion	1.049	0.045	0.265	0.400
Agreeableness	1.262	0.059	<0.001	<0.001
Low emotional stability	1.030	0.045	0.501	0.607
Cognitive ability	1.071	0.042	0.079	0.153
Age	2.251	0.183	<0.001	<0.001
Age^2^	0.982	0.002	<0.001	<0.001
Gender: Femalefemale (vs. male)	0.920	0.055	0.161	0.268
Gender: diverse (vs. male)	1.527	0.524	0.218	0.341
Gender: diverse (vs. female)	1.666	0.568	0.139	0.241
(3) I use public transport less often/not at all				
Predictor	*OR*	*SE*	*p*	*p*_*cor*_
Openness	0.995	0.049	0.924	0.924
Conscientiousness	1.048	0.049	0.310	0.451
Extraversion	1.066	0.044	0.121	0.214
Agreeableness	1.182	0.053	<0.001	0.001
Low emotional stability	1.162	0.049	<0.001	0.001
Cognitive ability	1.126	0.042	0.001	0.004
Age	1.863	0.148	<0.001	<0.001
Age^2^	0.986	0.002	<0.001	<0.001
Gender: female (vs. male)	1.037	0.058	0.508	0.607
Gender: diverse (vs. male)	1.305	0.403	0.389	0.536
Gender: diverse (vs. female)	1.257	0.386	0.457	0.571
(4) I avoid (larger) crowds				
Predictor	*OR*	*SE*	*p*	*p*_*cor*_
Openness	0.980	0.054	0.716	0.769
Conscientiousness	1.038	0.053	0.466	0.582
Extraversion	0.928	0.042	0.102	0.193
Agreeableness	1.320	0.065	<0.001	<0.001
Low emotional stability	1.025	0.048	0.595	0.690
Cognitive ability	1.141	0.048	0.001	0.004
Age	2.337	0.195	<0.001	<0.001
Age^2^	0.981	0.016	<0.001	<0.001
Gender: female (vs. male)	1.129	0.070	0.049	0.102
Gender: diverse (vs. male)	1.532	0.547	0.231	0.356
Gender: diverse (vs. female)	1.357	0.483	0.390	0.544
(5) I restrict meetings with family/friends as a precaution				
Predictor	*OR*	*SE*	*p*	*p*_*cor*_
Openness	0.954	0.051	0.381	0.534
Conscientiousness	0.963	0.048	0.457	0.580
Extraversion	1.021	0.045	0.640	0.718
Agreeableness	1.410	0.068	<0.001	<0.001
Low emotional stability	1.061	0.048	0.191	0.305
Cognitive ability	1.222	0.049	<0.001	<0.001
Age	2.337	1.928	<0.001	<0.001
Age^2^	0.981	0.016	<0.001	<0.001
Gender: female (vs. male)	1.103	0.067	0.103	0.192
Gender: diverse (vs. male)	1.728	0.599	0.114	0.208
Gender: diverse (vs. female)	1.567	0.541	0.194	0.277
(6) I have bought more supplies than usual				
Predictor	*OR*	*SE*	*p*	*p*_*cor*_
Openness	0.980	0.062	0.749	0.789
Conscientiousness	1.088	0.065	0.155	0.267
Extraversion	0.991	0.052	0.862	0.874
Agreeableness	0.941	0.054	0.297	0.440
Low emotional stability	1.322	0.071	<0.001	<0.001
Cognitive ability	1.029	0.050	0.558	0.656
Age	1.356	0.143	0.004	0.009
Age^2^	0.994	0.002	0.003	0.008
Gender: female (vs. male)	1.062	0.078	0.408	0.536
Gender: diverse (vs. male)	0.757	0.316	0.505	0.607
Gender: diverse (vs. female)	0.713	0.296	0.415	0.593
(7) I feel more insecure in public space than usual				
Predictor	*OR*	*SE*	*p*	*p*_*cor*_
Openness	0.876	0.047	0.014	0.031
Conscientiousness	1.235	0.063	<0.001	<0.001
Extraversion	0.991	0.044	0.845	0.867
Agreeableness	1.041	0.051	0.409	0.536
Low emotional stability	1.597	0.073	<0.001	<0.001
Cognitive ability	0.893	0.036	0.005	0.013
Age	1.472	0.132	<0.001	<0.001
Age^2^	0.992	0.018	<0.001	<0.001
Gender: female (vs. male)	1.092	0.068	0.156	0.267
Gender: diverse (vs. male)	1.738	0.526	0.068	0.138
Gender: diverse (vs. female)	1.591	0.479	0.122	0.175
(8) I expect financial losses (e.g. due to loss of my student job)				
Predictor	*OR*	*SE*	*p*	*p*_*cor*_
Openness	0.914	0.045	0.070	0.140
Conscientiousness	0.983	0.045	0.720	0.769
Extraversion	1.180	0.048	<0.001	<0.001
Agreeableness	1.017	0.046	0.715	0.769
Low emotional stability	1.270	0.053	<0.001	<0.001
Cognitive ability	0.878	0.033	<0.001	0.001
Age	2.024	0.165	<0.001	<0.001
Age^2^	0.986	0.002	<0.001	<0.001
Gender: female (vs. male)	0.867	0.049	0.011	0.026
Gender: diverse (vs. male)	1.499	0.441	0.169	0.276
Gender: diverse (vs. female)	1.729	0.507	0.062	0.087

*Note*. *OR* = Odds Ratio from logistic regressions; *SE* = standard error; *p* = *p*-value; *p*_cor_ = *p*-value, corrected for multiple testing using the by-method of Benjamini and Hochberg.

**Table 3:** Associations found between the Big Five personality traits and students’ thoughts, feelings, and behaviors during the first three weeks of the COVID-19 pandemic in Germany.

		O	C	E	A	Low ES
1	I consciously keep an eye on the news situation		+		+	
2	I wash my hands more often/intensively				+	
3	I use public transport less often/not at all				+	+
4	I avoid (larger) crowds				+	
5	I restrict meetings with family/friends as a precaution				+	
6	I have bought more supplies than usual					+
7	I feel more insecure in public space than usual	−	+			+
8	I expect financial losses (e.g. due to loss of my student job)			+		+

*Note*: O = Openness; C = Conscientiousness; E = Extraversion; A = Agreeableness; ES = low emotional stability. Only significant associations (p < 0.05 after correction for multiple testing) are considered.

### Control variables

With respect to cognitive abilities, our models revealed that individuals with higher cognitive abilities more often conscientiously kept up on the news (OR = 1.117). With one exception (washing hands more often/intensively), individuals with higher cognitive abilities also complied more often with governmental rules and recommendations (OR from 1.126 to 1.222). Additionally, higher cognitive abilities were unrelated to hoarding supplies and related to lower probabilities of feeling insecure in public space (OR = 0.893) and of expecting financial losses (OR = 0.878).

With regard to age, we found that older individuals more often experienced each of the examined thoughts, feelings, and behaviors (OR from 1.356 to 2.337). However, there were no gender differences, except that women expected financial losses less often compared to men (OR = 0.867). Note that the number of diverse individuals was quite low, hampering the detection of gender differences between women and diverse individuals as well as between male and diverse individuals.

Our study was conducted at the same time as public health measures were being introduced in Germany. Because participants could have completed the survey slightly before, during, or after their introduction, we repeated the analyses and included additional dummies for each day of answering the survey to control for timing effects. The estimated coefficients of these dummies (for the day the survey was taken) are presented in S1 Fig. However, regression analyses revealed no significant associations of these dummy variables with the respective outcomes.

### Personality

With respect to personality, we found that more conscientious (OR = 1.133) and more agreeable (OR = 1.285) individuals more often conscientiously kept up with the news. More agreeable individuals were also more likely to wash their hands more often/intensively (OR = 1.262), use public transport less (OR = 1.182), avoid crowds (OR = 1.320), and restrict meetings with family/friends (OR = 1.410). None of the other Big Five personality traits were associated with these behaviors, except that less emotionally stable individuals were also more likely to use public transport less (OR = 1.162). Taken together, more conscientious and more agreeable individuals, in particular, kept up with the news, and more agreeable individuals, in particular, complied with governmental rules and recommendations to prevent further spread of COVID-19.

At the same time, less emotionally stable individuals, in particular, were more likely to buy more supplies than usual (OR = 1.322), feel more insecure in public spaces than usual (OR = 1.597), and expect financial losses (OR = 1.270). Moreover, less open (OR = 0.876) and more conscientious (OR = 1.235) individuals more often felt insecure in public spaces, and more extraverted individuals more often expected financial losses (OR = 1.180). Taken together, less emotionally stable individuals, in particular, tended to hoard supplies, feel insecure, and fear financial losses due to the crisis.

## Discussion

How did students think, feel, and behave at the beginning of the COVID-19 pandemic in Germany? Our main findings are as follows: First, more agreeable individuals, in particular, kept up with the news and complied with governmental rules and recommendations. Other Big Five traits, however, were largely unrelated to these behaviors. Second, less emotionally stable individuals, in particular, hoarded supplies, felt less secure, and feared financial losses due to the crisis.

With regard to age, we found that older students more often experienced each of the examined thoughts, feelings, and behaviors. The developmental period of young adulthood is characterized by considerable change, including many age-graded major life events and transitions [28,29]. For example, comparatively young students often still live with their parents, whereas comparatively old students often have already started a family [30,31]. It is thus plausible to assume that students’ daily routines and activities vary substantially by age, which might explain our age effects on students’ thoughts, feelings, and behaviors during the COVID-19 pandemic. However, in unreported additional robustness analyses, we find that our main results on students’ thoughts, feelings, and behaviors during the COVID-19 pandemic are robust to the exclusion of very young or old students.

We found that less open individuals more often felt less secure in public spaces. This finding is inconsistent with our hypotheses, but could be explained by less open individuals tending to have greater difficulties adjusting to the new situation, which could lead to higher insecurity.

With respect to conscientiousness, our hypotheses were not confirmed, In contrast to previous research [3,4], there was no evidence that more conscientious people complied more strictly with governmental rules and recommendations. Instead, more conscientious individuals more often kept up with the news and felt insecure in public spaces.

In terms of extraversion, our study revealed that more extraverted individuals more often feared financial losses due to the crisis. This finding is consistent with our hypotheses and previous findings [4] and could be explained by the possibility that more extraverted people, in particular, tend to work in sectors hit by the crisis (e.g., the event industry) [32]. However, no evidence was found that more extraverted individuals were less likely to engage in social distancing, which is inconsistent with our hypotheses and other findings [3]. It is possible that highly extraverted people tend to be particularly creative in finding alternative ways to communicate with others (e.g., by phone or video chat), which could explain these results.

With regard to agreeableness, we found that more agreeable individuals more often kept up with the news and complied with governmental rules and recommendations. These findings confirm our hypotheses and previous evidence that greater agreeableness relates to higher engagement in containment measures [4]. One could speculate whether more agreeable people are more willing to behave in a socially desirable way, to maintain positive relationships with others. More agreeable individuals could also have greater empathy for vulnerable people and thus be more motivated to adhere to public health measures to protect them [7–9,33].

Finally, our study revealed that less emotionally stable individuals more often hoarded supplies, felt insecure, and feared financial losses due to the crisis. These results are consistent with our hypotheses and previous evidence linking lower emotional stability to higher financial concerns [4]. However, our assumption that less emotionally stable individuals adhere more strongly to governmental rules and recommendations was not confirmed. Less emotionally stable individuals could experience higher levels of fear, insecurity, and distress due to the COVID-19 pandemic. Therefore, they could feel more burdened and have lower capacities to engage in public health measures to protect themselves and others [34], which could explain these results.

### Strengths and limitations

A particular strength of our study is its large sample: We studied thousands of students just as COVID-19 became a serious public health concern in Germany, and asked for their thoughts, feelings, and behaviors due to the crisis. Personality was assessed with the IPIP, a well-established and comprehensive measure [24]. Our analyses were not only adjusted for sociodemographic factors and cognitive abilities, but also controlled for multiple testing.

Although the Fachkraft 2030 survey participants have been shown to be roughly comparable to the student population in Germany (e.g., in terms of sociodemographics) [23], the survey is not based on a representative sample. Thus, the generalizability of our findings to the entire student population in Germany, non-students, older adults, and people outside of Germany could be limited. Moreover, thoughts, feelings, and behaviors due to the crisis were assessed via self-reporting only. Whether the participants actually complied with specific rules and recommendations was not tested/observed.

## Conclusions

Our study suggests that more agreeable people, in particular, tend to adhere to public health rules and recommendations to fight COVID-19. At the same time, less emotionally stable individuals, in particular, often hoard supplies, feel insecure, and fear financial losses due to the crisis. Such thoughts, feelings, and behaviors could turn out to be problematic and result in a vicious cycle of distress. For example, fearful expectations about one’s future could turn into a self-fulfilling prophecy, and panic behaviors, such as hoarding supplies, can impede successful crisis management.

Our findings are of great relevance not only to basic psychological and economic sciences, but also to applied research on targeted interventions to fight COVID-19 and associated unfavorable consequences [11–13]. For example, less emotionally stable people, in particular, could benefit from targeted interventions to cope with distress, fear, and insecurity and thus prevent other unfavorable outcomes, such as hoarding supplies. Future research should not only replicate our results, but also examine such interventions.

## Supporting information

S1 Fig Development of how specific thoughts, feelings, and behaviors toward COVID-19 changed over time. (DOCX)

S1 Table German translations of the English version of the International Personality Item Pool (IPIP). (DOCX)

S2 Table Correlations between all predictor and outcome variables. (DOCX)
