# Supplementary material for: The role of personality in the thoughts, feelings, and behaviors of students in Germany during the first weeks of the COVID-19 pandemic
Source: PLoS One. 2020 Nov 30;15(11):e0242904. doi: 10.1371/journal.pone.0242904 (PMC7703888; doi:10.1371/journal.pone.0242904)
Supplement: S1 Fig — (DOCX) [file pone.0242904.s001.docx]

**Figure S1.**

Development of how specific thoughts, feelings, and behaviors toward COVID-19 changed over time

*Note:* Development of how specific thoughts, feelings, and behaviors toward COVID-19 changed over time (in days) relative to the first measurement day (black line). The grey lines mark the 95% confidence interval.
