# Supplementary material for: The role of personality in the thoughts, feelings, and behaviors of students in Germany during the first weeks of the COVID-19 pandemic
Source: PLoS One. 2020 Nov 30;15(11):e0242904. doi: 10.1371/journal.pone.0242904 (PMC7703888; doi:10.1371/journal.pone.0242904)
Supplement: S1 Table — (DOCX) [file pone.0242904.s002.docx]

**Table S1**

German translations of the English version of the International Personality Item Pool (IPIP)

|  | English original | German translation |
| --- | --- | --- |
|  | Extraversion |  |
| 1 | Am the life of the party. | Ich bringe Leben in eine Party. |
| 2 | Feel comfortable around people. | Ich fühle mich in Gesellschaft anderer wohl. |
| 3 | Start conversations. | Ich beginne Unterhaltungen. |
| 4 | Talk to a lot of different people at parties. | Auf Partys spreche ich mit vielen verschiedenen Leuten. |
| 5 | Don't mind being the center of attention. | Es stört mich nicht, im Mittelpunkt der Aufmerksamkeit zu stehen. |
| 6 | Don't talk a lot. | Ich rede nicht viel. |
| 7 | Keep in the background. | Ich halte mich im Hintergrund. |
| 8 | Have little to say. | Ich habe wenig zu sagen. |
| 9 | Don't like to draw attention to myself. | Ich ziehe nicht gern Aufmerksamkeit auf mich. |
| 10 | Am quiet around strangers. | Ich bin schweigsam unter fremden Menschen. |
|  |  |  |
|  | Agreeableness |  |
| 11 | Am interested in people. | Ich interessiere mich für Leute. |
| 12 | Sympathize with others' feelings. | Ich kann die Gefühle anderer verstehen. |
| 13 | Have a soft heart. | Ich habe ein weiches Herz. |
| 14 | Take time out for others. | Ich nehme mir Zeit für andere. |
| 15 | Feel others' emotions. | Ich kann die Gefühle anderer nachfühlen. |
| 16 | Make people feel at ease. | In meiner Gegenwart fühlen sich andere wohl. |
| 17 | Am not really interested in others. | Ich interessiere mich nicht wirklich für andere. |
| 18 | Insult people. | Ich beleidige Leute. |
| 19 | Am not interested in other people's problems. | Ich interessiere mich nicht für die Probleme anderer Leute. |
| 20 | Feel little concern for others. | Andere Menschen kümmern mich wenig. |
|  |  |  |
|  | Conscientiousness |  |
| 21 | Am always prepared. | Ich bin immer vorbereitet. |
| 22 | Pay attention to details. | Ich achte auf Details. |
| 23 | Get chores done right away. | Ich erledige Hausarbeit sofort. |
| 24 | Like order. | Ich mag Ordnung. |
| 25 | Follow a schedule. | Ich folge einem Plan. |
| 26 | Am exacting in my work. | Bei der Arbeit bin ich exakt. |
| 27 | Leave my belongings around. | Ich lasse meine Sachen herumliegen. |
| 28 | Make a mess of things. | Ich setze Dinge in den Sand. |
| 29 | Often forget to put things back in their proper place. | Ich vergesse oft, Dinge wieder an den richtigen Platz zurück zu legen. |
| 30 | Shirk my duties. | Ich drücke mich vor meinen Pflichten. |
|  |  |  |
|  | Emotional stability |  |
| 31 | Am relaxed most of the time. | Ich bin die meiste Zeit entspannt. |
| 32 | Seldom feel blue. | Ich fühle mich selten traurig. |
| 33 | Get stressed out easily. | Ich rege mich leicht auf. |
| 34 | Worry about things. | Ich mache mir Sorgen um Dinge. |
| 35 | Am easily disturbed. | Ich fühle mich schnell gestört. |
| 36 | Get upset easily. | Ich gerate leicht aus der Fassung. |
| 37 | Change my mood a lot. | Meine Laune ändert sich häufig. |
| 38 | Have frequent mood swings. | Ich habe häufig Stimmungsschwankungen. |
| 39 | Get irritated easily. | Ich lasse mich leicht irritieren. |
| 40 | Often feel blue. | Ich fühle mich oft traurig. |
|  |  |  |
|  | Openness to experience |  |
| 41 | Have a rich vocabulary. | Ich habe einen reichen Wortschatz. |
| 42 | Have a vivid imagination. | Ich habe eine lebhafte Vorstellungskraft. |
| 43 | Have excellent ideas. | Ich habe hervorragende Ideen. |
| 44 | Am quick to understand things. | Ich verstehe Dinge schnell. |
| 45 | Use difficult words. | Ich benutze schwierige Worte. |
| 46 | Spend time reflecting on things. | Ich verbringe Zeit damit, Dinge zu reflektieren. |
| 47 | Am full of ideas. | Ich bin voller Ideen. |
| 48 | Have difficulty understanding abstract ideas. | Ich habe Schwierigkeiten abstrakte Ideen zu verstehen. |
| 49 | Am not interested in abstract ideas. | Ich interessiere mich nicht für abstrakte Ideen. |
| 50 | Do not have a good imagination. | Ich habe keine gute Vorstellungskraft. |
