# Supplementary material for: The role of personality in the thoughts, feelings, and behaviors of students in Germany during the first weeks of the COVID-19 pandemic
Source: PLoS One. 2020 Nov 30;15(11):e0242904. doi: 10.1371/journal.pone.0242904 (PMC7703888; doi:10.1371/journal.pone.0242904)
Supplement: S2 Table — (DOCX) [file pone.0242904.s003.docx]

**Table S2**

Correlations between all predictor and outcome variables

|  | Variables | (1) | (2) | (3) | (4) | (5) | (6) | (7) | (8) | (9) | (10) | (11) | (12) | (13) | (14) | (15) | (16) | (17) | (18) | (19) | (20) |
| --- | --- | --- | --- | --- | --- | --- | --- | --- | --- | --- | --- | --- | --- | --- | --- | --- | --- | --- | --- | --- | --- |
| (1) | Following news | 1.000 |  |  |  |  |  |  |  |  |  |  |  |  |  |  |  |  |  |  |  |
|  |  |  |  |  |  |  |  |  |  |  |  |  |  |  |  |  |  |  |  |  |  |
| (2) | Washing hands | 0.500 | 1.000 |  |  |  |  |  |  |  |  |  |  |  |  |  |  |  |  |  |  |
|  |  | (0.000) |  |  |  |  |  |  |  |  |  |  |  |  |  |  |  |  |  |  |  |
| (3) | Restricting meetings | 0.497 | 0.543 | 1.000 |  |  |  |  |  |  |  |  |  |  |  |  |  |  |  |  |  |
|  |  | (0.000) | (0.000) |  |  |  |  |  |  |  |  |  |  |  |  |  |  |  |  |  |  |
| (4) | Avoiding larger crowds | 0.554 | 0.605 | 0.663 | 1.000 |  |  |  |  |  |  |  |  |  |  |  |  |  |  |  |  |
|  |  | (0.000) | (0.000) | (0.000) |  |  |  |  |  |  |  |  |  |  |  |  |  |  |  |  |  |
| (5) | Avoiding public transport | 0.428 | 0.464 | 0.552 | 0.582 | 1.000 |  |  |  |  |  |  |  |  |  |  |  |  |  |  |  |
|  |  | (0.000) | (0.000) | (0.000) | (0.000) |  |  |  |  |  |  |  |  |  |  |  |  |  |  |  |  |
| (6) | Prepping behavior | 0.201 | 0.208 | 0.194 | 0.200 | 0.209 | 1.000 |  |  |  |  |  |  |  |  |  |  |  |  |  |  |
|  |  | (0.000) | (0.000) | (0.000) | (0.000) | (0.000) |  |  |  |  |  |  |  |  |  |  |  |  |  |  |  |
| (7) | Feeling insecure in public space | 0.269 | 0.311 | 0.285 | 0.320 | 0.323 | 0.240 | 1.000 |  |  |  |  |  |  |  |  |  |  |  |  |  |
|  |  | (0.000) | (0.000) | (0.000) | (0.000) | (0.000) | (0.000) |  |  |  |  |  |  |  |  |  |  |  |  |  |  |
| (8) | Expecting financial losses | 0.218 | 0.267 | 0.267 | 0.284 | 0.231 | 0.131 | 0.172 | 1.000 |  |  |  |  |  |  |  |  |  |  |  |  |
|  |  | (0.000) | (0.000) | (0.000) | (0.000) | (0.000) | (0.000) | (0.000) |  |  |  |  |  |  |  |  |  |  |  |  |  |
| (9) | Openness | 0.020 | -0.009 | 0.022 | 0.012 | 0.021 | -0.013 | -0.038 | -0.023 | 1.000 |  |  |  |  |  |  |  |  |  |  |  |
|  |  | (0.091) | (0.442) | (0.068) | (0.298) | (0.082) | (0.270) | (0.002) | (0.057) |  |  |  |  |  |  |  |  |  |  |  |  |
| (10) | Conscientiousness | 0.051 | 0.014 | 0.014 | 0.029 | 0.022 | 0.002 | 0.025 | -0.023 | 0.216 | 1.000 |  |  |  |  |  |  |  |  |  |  |
|  |  | (0.000) | (0.251) | (0.230) | (0.015) | (0.073) | (0.877) | (0.038) | (0.056) | (0.000) |  |  |  |  |  |  |  |  |  |  |  |
| (11) | Extraversion | 0.023 | 0.022 | 0.023 | -0.003 | 0.024 | -0.021 | -0.032 | 0.029 | 0.290 | 0.113 | 1.000 |  |  |  |  |  |  |  |  |  |
|  |  | (0.059) | (0.067) | (0.053) | (0.790) | (0.048) | (0.083) | (0.008) | (0.015) | (0.000) | (0.000) |  |  |  |  |  |  |  |  |  |  |
| (12) | Agreeableness | 0.088 | 0.069 | 0.110 | 0.090 | 0.069 | -0.012 | 0.011 | -0.004 | 0.289 | 0.234 | 0.294 | 1.000 |  |  |  |  |  |  |  |  |
|  |  | (0.000) | (0.000) | (0.000) | (0.000) | (0.000) | (0.337) | (0.339) | (0.751) | (0.000) | (0.000) | (0.000) |  |  |  |  |  |  |  |  |  |
| (13) | Low emotional stability | 0.002 | 0.006 | 0.019 | 0.018 | 0.042 | 0.070 | 0.134 | 0.060 | -0.103 | -0.195 | -0.221 | -0.076 | 1.000 |  |  |  |  |  |  |  |
|  |  | (0.852) | (0.639) | (0.122) | (0.140) | (0.000) | (0.000) | (0.000) | (0.000) | (0.000) | (0.000) | (0.000) | (0.000) |  |  |  |  |  |  |  |  |
| (14) | Cognitive ability | 0.041 | 0.022 | 0.072 | 0.048 | 0.046 | 0.006 | -0.038 | -0.049 | 0.101 | 0.020 | -0.037 | 0.045 | -0.037 | 1.000 |  |  |  |  |  |  |
|  |  | (0.001) | (0.064) | (0.000) | (0.000) | (0.000) | (0.646) | (0.002) | (0.000) | (0.000) | (0.095) | (0.002) | (0.000) | (0.002) |  |  |  |  |  |  |  |
| (15) | Gender: Male | -0.034 | -0.025 | -0.067 | -0.070 | -0.051 | -0.027 | -0.069 | 0.012 | 0.028 | -0.118 | 0.005 | -0.265 | -0.242 | -0.001 | 1.000 |  |  |  |  |  |
|  |  | (0.004) | (0.039) | (0.000) | (0.000) | (0.000) | (0.025) | (0.000) | (0.316) | (0.021) | (0.000) | (0.690) | (0.000) | (0.000) | (0.921) |  |  |  |  |  |  |
| (16) | Gender: Female | 0.035 | 0.023 | 0.065 | 0.068 | 0.050 | 0.028 | 0.064 | -0.015 | -0.032 | 0.120 | 0.002 | 0.268 | 0.234 | 0.000 | -0.985 | 1.000 |  |  |  |  |
|  |  | (0.003) | (0.059) | (0.000) | (0.000) | (0.000) | (0.020) | (0.000) | (0.203) | (0.007) | (0.000) | (0.887) | (0.000) | (0.000) | (0.985) | (0.000) |  |  |  |  |  |
| (17) | Gender: Diverse | -0.008 | 0.012 | 0.010 | 0.008 | 0.007 | -0.007 | 0.023 | 0.019 | 0.028 | -0.013 | -0.038 | -0.024 | 0.042 | 0.006 | -0.066 | -0.106 | 1.000 |  |  |  |
|  |  | (0.497) | (0.313) | (0.387) | (0.498) | (0.584) | (0.578) | (0.053) | (0.114) | (0.022) | (0.273) | (0.002) | (0.049) | (0.001) | (0.641) | (0.000) | (0.000) |  |  |  |  |
| (18) | Age | -0.128 | -0.152 | -0.151 | -0.173 | -0.135 | -0.013 | -0.056 | 0.015 | -0.012 | -0.026 | -0.006 | -0.065 | -0.047 | -0.009 | 0.107 | -0.107 | 0.002 | 1.000 |  |  |
|  |  | (0.000) | (0.000) | (0.000) | (0.000) | (0.000) | (0.269) | (0.000) | (0.207) | (0.300) | (0.032) | (0.592) | (0.000) | (0.000) | (0.469) | (0.000) | (0.000) | (0.836) |  |  |  |
| (19) | Age^2^ | -0.137 | -0.164 | -0.164 | -0.186 | -0.145 | -0.016 | -0.060 | 0.006 | -0.011 | -0.028 | -0.005 | -0.067 | -0.047 | -0.012 | 0.110 | -0.111 | 0.004 | 0.996 | 1.000 |  |
|  |  | (0.000) | (0.000) | (0.000) | (0.000) | (0.000) | (0.173) | (0.000) | (0.590) | (0.366) | (0.020) | (0.673) | (0.000) | (0.000) | (0.302) | (0.000) | (0.000) | (0.755) | (0.000) |  |  |
| (20) | Time | -0.098 | -0.047 | 0.056 | -0.030 | 0.041 | -0.030 | -0.009 | 0.005 | -0.009 | -0.015 | 0.006 | 0.003 | -0.005 | 0.000 | 0.018 | -0.016 | -0.011 | 0.075 | 0.074 | 1.000 |
|  |  | (0.000) | (0.000) | (0.000) | (0.012) | (0.001) | (0.014) | (0.442) | (0.691) | (0.467) | (0.225) | (0.589) | (0.808) | (0.702) | (0.971) | (0.134) | (0.183) | (0.350) | (0.000) | (0.000) |  |

*Note:* Pearson correlations. The *p*-values are in parentheses. Three binary gender variables were created to denote men versus all others, women versus all others, and diverse versus all others, respectively.
